# Serum 25-Hydroxyvitamin D Concentrations Are Associated with Mental Health and Psychosocial Stress in Young Adults

**DOI:** 10.3390/nu12071938

**Published:** 2020-06-30

**Authors:** Li Chen, Haidong Zhu, Gregory A. Harshfield, Frank A. Treiber, Jennifer S. Pollock, David Pollock, Olivia I. Okereke, Shaoyong Su, Yanbin Dong

**Affiliations:** 1 Georgia Prevention Institute, Department of Medicine, Medical College of Georgia, Augusta University, Augusta, GA 30912, USA; lichen1@augusta.edu (L.C.); hzhu@augusta.edu (H.Z.); GHARSHFI@augusta.edu (G.A.H.); ssu@augusta.edu (S.S.); 2 Department of Nursing Operating, College of Nursing, Medical University of South Carolina, Charleston, SC 29425, USA; treiberf@musc.edu; 3 College of Medicine, Medical University of South Carolina, Charleston, SC 29425, USA; 4 Section of Cardio-Renal Physiology and Medicine, Department of Medicine, Division of Nephrology, The University of Alabama at Birmingham, Birmingham, AL 35233, USA; jenniferpollock@uabmc.edu (J.S.P.); davidpollock@uabmc.edu (D.P.); 5 Department of Psychiatry, Massachusetts General Hospital and Harvard Medical School, Boston, MA 02114, USA; Olivia.Okereke@MGH.HARVARD.EDU

**Keywords:** 25-hydroxyvitamin D_3_, depression, anxiety, perceived stress, hostility

## Abstract

We aimed to test the hypothesis that serum 25-hydroxyvitamin D_3_ (25(OH)D) concentration is associated with mental health and life stress measures in young adults and investigate gender and racial disparities in these associations. This study comprised 327 black and white participants. Depression, trait anxiety, perceived stress, and hostility were measured by the following validated instruments: Beck Depression Inventory (BDI), State-Trait Anxiety Inventory (STAI), Perceived Stress Scale (PSS), and Cook–Medley Hostility Scale (CMHS). Linear regression was used to estimate correlations between serum 25(OH)D concentration and mental health measurements in the total population and in subgroups stratified by gender and race. In this sample (28.2 ± 3.1 years, 52% female, 53% black), serum 25(OH)D concentration was negatively related to BDI, STAI, PSS, total CMHS score, and the majority of CMHS subscale scores (*p*-values < 0.05). Stratified by gender, most of these associations remained significant only in women (*p*-values < 0.05). Stratified by race, higher 25(OH)D concentrations in white participants were significantly related to lower BDI, STAI, PSS, and CMHS-cynicism subscales (*p*-values < 0.05); 25(OH)D concentrations in the black participants were only inversely associated with CMHS and most CMHS subscales (*p*-values < 0.05) but not with BDI, STAI, and PSS. We present novel findings of consistent inverse relationships between serum 25(OH)D concentration and various measures of mental health and life stress. Long-term interventional studies are warranted in order to investigate the roles of vitamin D supplementation in the prevention and mitigation of depression, anxiety, and psychological stress in young adults.

## 1. Introduction

Mental health is an important dimension of health, affecting overall quality of life, and mental disorders impose a significant economic, social, and public health burden [1]. Mental disorders not only compromise patients’ quality of life but are also important risk factors for physical/somatic diseases. Studies suggest that depression is associated with inflammation [2] and cardiovascular disease [3]. Higher perceived stress has been related to worse outcomes of aging [4] and immunity [5]. In addition to depression, other areas of mental health and stress can affect disease. For example, anxiety was significantly associated with coronary artery calcification [6], and hostility is related to insulin resistance and inflammation [7].

Vitamin D is now increasingly recognized for its effects on mental health [8]. Vitamin D receptors are present in multiple brain regions, which suggests that vitamin D may have autocrine/paracrine properties in the human brain [9]. Vitamin D has also been shown to increase the synthesis of neurotransmitters, including dopamine and norepinephrine, in rats [10]. Depression, anxiety, perceived stress, and hostility are the key negative emotions that could affect cardiovascular health and mortality [11,12]. Therefore, the associations of vitamin D with those negative emotions would offer novel perspectives in cardiovascular health management.

Many studies which examined plasma levels of vitamin D and their association with mental health disorders have focused upon depression. The outcomes of these studies have been mixed. An inverse association between circulating vitamin D concentration and depression has been observed in adults in New Zealand [13], Australia [14], Finland [15], and the elderly in the U.S. [16]. Other studies found that the association was not statistically significant among the elderly in Italy [17] and the Netherlands [18], or among adults in Denmark [19] and the U.S. [20]. A large, randomized, controlled trial (RCT) comprising 36,282 postmenopausal women in the U.S. showed no significant association between 400 IU/day vitamin D supplementation and depression symptoms [21].

The relationship between serum 25(OH)D concentration and anxiety symptoms has been assessed in a few studies, and the associations found were mostly insignificant [14,15,19,22]. However, these findings may be limited by the fact that, in most studies, the anxiety measures were limited and typically were subscales of depression used in questionnaires [14,15,19,22,23]. The association between vitamin D and perceived stress or hostility is also under-studied. Only one study found serum 25(OH)D concentration to be inversely associated with perceived stress. The study involved Korean female elders, and perceived stress was characterized as a dummy variable based on a single question [24].

The majority of the studies conducted to date have involved the elderly, and evidence is scarce in young adults, especially in the United States. Therefore, we aimed to test the hypothesis that serum 25(OH)D concentration is associated with mental health and life stress measurements including depression, trait anxiety, perceived stress, and hostility in young adults. Moreover, previous studies have found a race difference in the effects of vitamin D on health outcomes [25,26], and sex hormones are closely related to vitamin D status [27]. Therefore, we also aimed to investigate gender and race disparities in these associations.

## 2. Materials and Methods

### 2.1. Participants

The recruitment and evaluation of participants for this cross-sectional study were part of a longitudinal cohort and have been described in detail elsewhere [28,29,30,31]. Briefly, participants were identified with family health history questionnaires obtained from a county-wide (Richmond County, Georgia) public school screening. Participants who met the following criteria were recruited: (1) aged 7 to 16 years in 1989; (2) black (African Americans) or white (European Americans); (3) normotensive for age and gender based on BP screening; and (4) apparently healthy based on parental reports of the child’s medical history. Among the follow-up visits, 327 serum samples collected on visit 15 between 2008 and 2010 were measured for serum 25(OH)D concentrations, which were used for the correlational analyses between the vitamin D status and mental health. The Institutional Review Board at Augustine University gave approval for the study (IRB identification code is 611084–t10), and the informed consent of each participant was obtained.

### 2.2. Vitamin D Measurement

Serum 25(OH)D concentrations were determined from fasting blood using an enzyme immunoassay (Immunodiagnostic Systems, Fountain Hills, AZ, USA), according to the manufacturer’s specifications. The analytical reliability of the 25(OH)D assays was monitored through participation in DEQAS (Vitamin D External Quality Assessment Scheme) and was deemed acceptable. The intra- and interassay coefficients of variation for serum 25(OH)D were 5.6% and 6.6%, respectively.

### 2.3. Beck Depression Inventory

The Beck Depression Inventory (BDI) was originally developed in 1961 to identify the presence and severity of depressive symptoms [32]. It has been well validated in normal and psychiatric populations [33]. All subjects were required to complete the self-report questionnaire, which contains 21 items. These items relate to feelings of irritability, guilt, and punishment, and physical symptoms such as fatigue, weight loss, and lack of interest in sex. Each item is rated on a 4-point Likert-type scale and scored on a scale value of 0 to 3. Theoretically, the total score of BDI can range from 0 to 63, and higher scores indicate more severe depressive symptoms.

### 2.4. State-Trait Anxiety Inventory

Anxiety was measured using the State-Trait Anxiety Inventory (STAI) from Y-2 [34]. Anxiety can be differentiated into state anxiety and trait anxiety. STAI is able to differentiate between participants suffering from anxiety and those suffering from depression. In this study, we measured trait anxiety, which is considered an enduring characteristic that refers to relatively stabilizing individual differences that characterize people’s anxiety or a general feeling of anxiety [34]. This inventory has 20 items. All items are rated on a 4-point Likert scale weighted from one to four. The total score for trait anxiety is the total of each item. Higher scores indicate greater anxiety.

### 2.5. Perceived Stress Scale

Perception of stress was measured by the Perceived Stress Scale (PSS), which contains 10 items, and each item was rated from zero to four using a 5-point Likert scale [35]. PSS is one of the most widely used psychological instruments for measuring nonspecific perceived stress.

### 2.6. Cook–Medley Hostility Scale

The Cook–Medley Hostility Scale (CMHS) is a 50-item, true/false questionnaire derived from the Minnesota Multiphasic Personality Inventory [36]. CMHS measures tendencies toward cynicism, hostile affect, and aggressive responding. Five subscales were identified: Cynicism, hostile attributions, hostile affect, aggressive responding, and social avoidance.

### 2.7. Hollingshead Social Status

The Hollingshead Four-Factor Social Status Index was calculated on the basis of parental education level, employment status, and occupation; a higher value indicates higher socioeconomic status (SES) [37]. The Hollingshead parental education level is rated on a 7-point scale that lists the highest grade completed, and the occupational prestige is rated on a 9-point scale. SES was then calculated by multiplying the occupation scale value by a weight of 5 and the education scale value by 3 and summing the products. Hollingshead Index scores range from 8 to 66.

### 2.8. Statistical Analysis

The general characteristics of the subjects are presented as mean ± standard deviation (SD) for continuous variables and N (%) for categorical variables. The normality of each continuous variable was tested based on a combined statistics test of skewness and kurtosis. To test mean differences in continuous measures by gender and race, an analysis-of-variance (ANOVA) was conducted for variables with normal distribution, while the Kruskal–Wallis test was used instead for non-normally distributed variables. Chi-square tests were conducted for categorical variables. Linear regression was used to estimate associations of psychological and psychosocial stress measures with 25(OH)D concentrations while adjusting for age, race, and gender in the base model and then further adjusting for SES in a separate model. Regression models were then stratified by gender and race to examine the differences by strata in the associations between 25(OH)D concentrations and the various mental health and stress-related scores. In order to investigate the item reliability of the psychosocial stress instruments, we assessed their internal consistency using Cronbach alpha indices. A two-sided *p*-value < 0.05 was considered statistically significant. All statistical analyses were performed using Stata version 12.0 (College Station, TX 77845 USA).

## 3. Results

### 3.1. Participant Characteristics

Among the 327 participants (28.2 ± 3.1 years, 52% female, 53% black), the mean serum 25(OH)D concentration was 59.2 ± 26.1 nmol/L. We compared general characteristics among the four groups, defined by gender and race. Age, 25(OH)D concentration, SES, and CHMS among the four groups were significantly different (*p*-value < 0.05) (Table 1). The Cronbach alpha indices for BDI, STAI, PSS, and CMHS are 0.9051, 0.9067, 0.8700, and 0.8994, respectively.

### 3.2. Adjusted Associations between Mental Health Measurements and Serum 25(OH)D Concentrations

In the base models, as shown in Table 2, higher serum 25(OH)D concentrations were significantly related to lower scores on the BDI, STAI, PSS, CMHS, and three CMHS subscales (*p*-values < 0.05) after adjustment for age, gender, and race; the aggressive responding (*p*-value = 0.067) and social aversion (*p*-value = 0.094) CMHS subscales were not related to 25(OH)D. Further adjustment for SES did not alter the findings significantly, but the association between 25(OH)D and the aggressive responding subscale became modestly significant (*p*-value = 0.045).

### 3.3. Gender Differences in the Associations between Mental Health Measurements and Serum 25(Oh)D Concentrations

**Figure 1:**
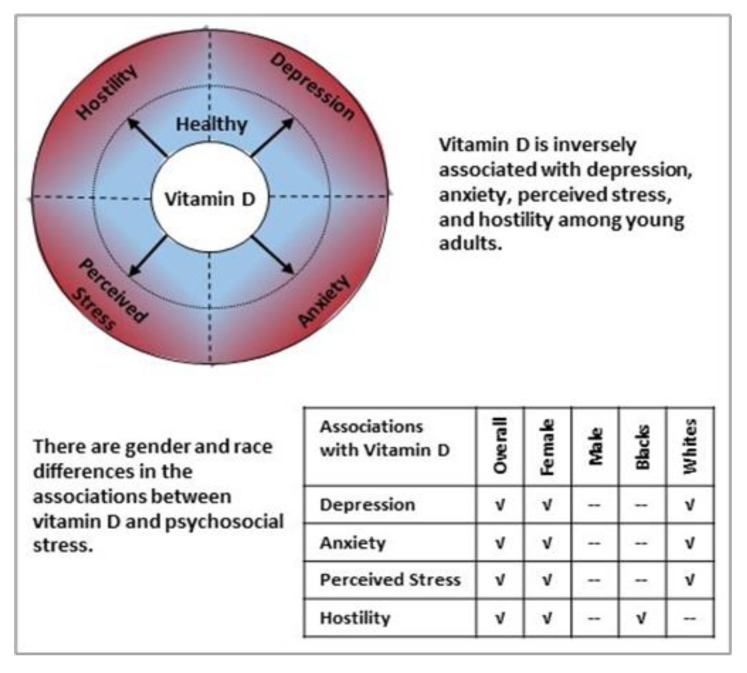
Summarization of the key findings.

Serum 25(OH)D concentrations were significantly related to lower scores on the BDI, STAI, PSS, CMHS, and all subscales of CMHS among females (*p*-values < 0.05) when adjusted for age and race. However, among males, serum 25(OH)D concentrations were only significantly associated with cynicism (*p*-value < 0.05). Further adjustment for SES did not change these findings. (Table 3, Figure 1).

### 3.4. Race Differences in the Associations between Mental Health Measurements and Serum 25(OH)D Concentrations

Among white participants, serum 25(OH)D concentrations were significantly related to lower scores on the BDI, STAI, PSS, and cynicism of CMHS (*p*-values < 0.05) after adjustment for age and gender. Among black participants, serum 25(OH)D concentrations were associated with most CMHS subscales (cynicism, hostile affect, aggressive responding, and hostile attribution (*p*-values < 0.05)) but were not related to depression, anxiety, or perceived stress. Further adjustment for SES did not change most findings, but the estimate for the association between 25(OH)D and hostile affect among black participants was attenuated and no longer statistically significant. (Table 4, Figure 1).

## 4. Discussion

In this study, we found that among U.S. young adults, serum 25(OH)D concentrations were significantly related to lower scores on the BDI, STAI, PSS, CMHS, and most CMHS subscales (except social aversion) after accounting for age, gender, race, and SES.

The associations between 25(OH)D concentration and the mental health scales were generally consistent across different mental health domains. Therefore, it is possible that these mental health domains may partially share common pathways linking them to vitamin D levels. Vitamin D receptors (VDR) and the vitamin D activating enzyme 1α-hydroxylase are present in the human brain [9], and VDR knockout mice have exhibited depressive behaviors [38]. Evidence also shows that 25(OH)D can cross the blood–brain barrier, and 25(OH)D receptors exist in the central nervous system (CNS) [39]. Vitamin D may also relate to mental health through the regulation of the levels of brain-derived neurotrophic factor (BDNF), which is a protein encoded by the *BDNF* gene. BDNF deficiency has been associated with age-dependent impairment in spatial learning [40], neurodegeneration [41], and cognitive dysfunction [42], as well as with depression [43,44,45]. Vitamin D supplementation may increase BDNF concentration in the hippocampus [46]—A critical brain region for emotion regulation. Neural stem cells treated with vitamin D have shown increased expression of *BDNF* [47].

Inflammation is correlated with mental health. Proinflammatory cytokines can induce depressive symptoms by affecting neurotransmitter metabolism, impairing neuronal health, and altering brain activity in mood-relevant brain regions [48]. Vitamin D may have a positive influence on human mental health through its regulatory effects on systemic inflammation. A review article based on five large cross-sectional studies found significant inverse associations between 25(OH)D and inflammation markers in persons with low 25(OH)D concentrations and in adults with high inflammation levels [49]. Another review noted that vitamin D supplementation can robustly reduce TNF-α and IL-6, but not other cytokines, yet TNF-α and IL-6 were the two cytokines that were the most robustly associated with depression in a meta-analysis [50]. Similarly, stress and hostility have also been positively correlated with TNF-α and IL-6 [51,52].

Oxidative stress has been linked to both depression and anxiety. Oxidative stress is increased in depressive subjects [53,54] and has been shown to increase anxiety in animal behavioral models [55]. Indeed, it has been proposed that the products of oxidative stress have the potential to serve as parameters for the measurement and prediction of depression status, as well as for determining the effectiveness of administrated antidepressants [56]. Vitamin D supplementation has also been related to reduced oxidative stress markers in several human studies [57]. Vitamin D treatment is able to reduce the apoptosis-related gene expression and prevent the loss of mitochondrial potential and the consequent cytochrome C release and caspase activation [58].

In the current study, inverse associations between serum 25(OH)D concentration and psychological and psychosocial measures were significant only among females when gender-stratified analyses were conducted. Several other studies observed similar findings [17]. For example, Toffanello ED et al. found that serum 25(OH)D concentration correlated inversely with the Geriatric Depression Scale only among women [17]. Mieun Gwon et al. found that serum 25(OH)D concentration was significantly associated with less perceived stress among Korean female older adults, while this association was not significant among male participants [24].

In addition to gender differences, we also observed racial differences in the associations between mental health and serum 25(OH)D concentrations. Lower levels of 25(OH)D were associated with increased depression, anxiety, and stress among white participants but were more associated with hostility measures among black participants. These differences suggest possible racial differences in the burden or distribution of different mental health or symptom variables. Nevertheless, measurement-related factors (e.g., differential item functioning, or item bias, in racial scales) cannot be excluded. Historically, the reporting of depression may have been stigmatized among black participants [59,60]; thus, seen in this context, experiences of hostility or hostile attribution might be considered more sensitive indicators of problems in the mental health domain. However, while older literature [60] has suggested this possibility, more recent work has shown that, in fact, a higher burden of depressive symptoms may be present among black compared to white adults, especially among older adults [61]. Therefore, it is clear overall that further research is required to address racial differences and disparities in mood outcomes and predictors therein.

To the best of our knowledge, this study is the first to analyze the association between serum 25(OH)D concentration and measures of hostility. Firstly, the study included a range of psychological and psychosocial measures that were specific to their respective domains; this is in contrast, for example, to prior studies that may have relied on capturing anxiety using subscales of depression in questionnaires [14,15,19,22] or measuring perceived stress with one question [24]. Secondly, this study focused on young adults, which is the age group at the highest risk for new onset of mental disorders. Thus, identifying modifiable predictors of adverse psychological and psychosocial status has direct relevance to public health. Thirdly, this study consisted of roughly equal distributions of different races and genders, which enabled us to study race and gender differences. The limitations of this study should also be noted. Firstly, this observational study cannot establish a causal relationship between serum 25(OH)D concentration and mental health. Secondly, we cannot exclude possible confounding by unmeasured factors (e.g., smoking, physical activity) that may be related to vitamin D and/or mood status due to the limited variables collected. It is hoped that results from long-term randomized trials with the experimental control of vitamin D levels using supplementation can shed more light on causal associations of 25(OH)D with mental health.

## 5. Conclusions

We observed consistent relationships between lower serum 25(OH)D concentrations and various measures of psychosocial stress and poorer mental health in a large sample of community-based young adults. Future work might address whether vitamin D supplementation and the optimization of 25(OH)D levels could be potential modalities for the prevention and/or amelioration of psychological distress in young persons.

## Figures and Tables

**Table 1:** General characteristics stratified by gender and race.

Characteristics	White Male (*n* = 83)	Black Male (*n* = 75)	White Female (*n* = 70)	Black Female (*n* = 99)	Total (*n* = 327)	*p*-Values
Age (years)	27.3 ± 3.1	28.7 ± 3.2	28.4 ± 3.2	28.3 ± 2.8	28.2 ± 3.1	0.031
25(OH)D (nmol/L)	74.7 ± 24.7	46.5 ± 18.1	76.0 ± 26.8	43.9 ± 15.3	59.2 ± 26.1	<0.001
SES	41.5 ± 15.8	37.9 ± 15.3	43.2 ± 14.5	34.9 ± 13.9	39.0 ± 15.2	0.001
BDI	6.4 ± 7.4	6.9 ± 8.2	6.1 ± 6.3	7.0 ± 7.9	6.6 ± 7.5	0.848
STAI	24.3 ± 5.9	24.6 ± 6.0	24.0 ± 6.7	24.9 ± 6.1d	24.5 ± 6.1	0.580
PSS	15.2 ± 6.5	15.7 ± 6.3	15.8 ± 7.5	16.4 ± 6.5	15.8 ± 6.7	0.691
CMHS	21.6 ± 9.2	24.6 ± 8.2	16.0 ± 8.8	18.4 ± 9.7	20.1 ± 9.5	<0.001
CMHS cynicism	6.4 ± 3.1	7.8 ± 3.1	4.2 ± 2.9	5.4 ± 3.6	5.9 ± 3.4	<0.001
CMHS hostile affect	2.1 ± 1.3	2.2 ± 1.3	1.7 ± 1.3	1.8 ± 1.3	1.9 ± 1.3	0.088
CMHS aggressive responding	4.0 ± 2.0	4.3 ± 1.7	2.7 ± 2.0	3.2 ± 2.1	3.6 ± 2.0	<0.001
CMHS hostile attribution	4.5 ± 2.7	5.7 ± 2.7	3.5 ± 2.4	4.1 ± 2.5	4.4 ± 2.7	<0.001
CMHS social aversion	1.8 ± 1.2	2.0 ± 1.1	1.7 ± 1.0	1.7 ± 1.1	1.8 ± 1.1	0.332

25(OH)D = 25-hydroxyvitamin D_3_; BDI = Beck Depression Inventory; STAI = State-Trait Anxiety Inventory; PSS = Perceived Stress Scale; CMHS = Cook–Medley Hostility Scale; SES = socioeconomic status.

**Table 2:** Adjusted associations between mental health measurements and serum 25(OH)D concentrations.

Dependent Variables	Base Model *		Base Model + SES *	
	β (SE)	*p*	β (SE)	*p*
BDI **	−0.16 (0.07)	0.018	−0.17 (0.07)	0.012
STAI ***	−0.19 (0.07)	0.005	−0.20 (0.07)	0.003
**PSS**	−0.20 (0.07)	0.004	−0.21 (0.07)	0.002
**CMHS**	−0.20 (0.06)	0.002	−0.22 (0.06)	0.001
Cynicism	−0.23 (0.06)	<0.001	−0.24 (0.06)	<0.001
Hostile affect	−0.16 (0.07)	0.022	−0.16 (0.07)	0.015
Aggressive responding	−0.12 (0.07)	0.067	−0.13 (0.07)	0.045
Hostile attribution	−0.19 (0.07)	0.005	−0.19 (0.07)	0.003
Social aversion	−0.12 (0.07)	0.094	−0.12 (0.07)	0.071

25(OH)D = 25-hydroxyvitamin D_3_; BDI = Beck Depression Inventory; STAI = State-Trait Anxiety Inventory; PSS = Perceived Stress Scale; CMHS = Cook–Medley Hostility Scale; SES = socioeconomic status. * Serum 25(OH)D was log-transformed and adjusted for age, gender, and race in the base model. Standardized β coefficients are presented. ** Square root-transformed. *** Log-transformed.

**Table 3:** Adjusted associations between mental health and serum 25(OH)D concentrations stratified by gender.

Dependent Variable	Male * (*n* = 158)				Female * (*n* = 169)			
	Base Model		Base Model + SES		Base Model		Base Model + SES	
	β (SE)	*p*	β (SE)	*p*	β (SE)	*p*	β (SE)	*p*
BDI **	−0.07 (0.10)	0.478	−0.05 (0.09)	0.563	−0.26 (0.09)	0.006	−0.22 (0.09)	0.013
STAI ***	−0.14 (0.09)	0.138	−0.13 (0.09)	0.142	−0.25 (0.10)	0.015	−0.21 (0.09)	0.028
**PSS**	−0.08 (0.09)	0.399	−0.08 (0.09)	0.376	−0.33 (0.10)	0.001	−0.29 (0.10)	0.03
**CMHS**	−0.06 (0.09)	0.483	−0.05 (0.08)	0.506	−0.38 (0.09)	<0.001	−0.35 (0.09)	<0.001
Cynicism	−0.23 (0.08)	0.007	−0.23 (0.08)	0.007	−0.24 (0.10)	0.012	−0.22 (0.09)	0.018
Hostile affect	0.00 (0.09)	0.994	0.02 (0.09)	0.868	−0.34 (0.10)	0.001	−0.31 (0.09)	0.001
Aggressive responding	0.04 (0.09)	0.648	0.05 (0.08)	0.559	−0.31 (0.10)	0.002	−0.29 (0.10)	0.003
Hostile attribution	−0.01 (0.10)	0.905	−0.00 (0.09)	0.984	−0.40 (0.09)	<0.001	−0.36 (0.08)	<0.001
Social aversion	0.09 (0.10)	0.376	0.09 (0.10)	0.343	−0.34 (0.09)	<0.001	−0.34 (0.09)	<0.001

25(OH)D = 25-hydroxyvitamin D_3_; BDI = Beck Depression Inventory; STAI = State-Trait Anxiety Inventory; PSS = Perceived Stress Scale; CMHS = Cook–Medley Hostility Scale; SES = socioeconomic status. * Serum 25(OH)D was log-transformed and adjusted for age and race in the base model. BDI was square root-transformed and STAI was log-transformed in the regression. Standardized β coefficients are presented. ** Square root-transformed. *** Log-transformed.

**Table 4:** Adjusted associations between mental health and serum 25(OH)D concentrations stratified by race.

Dependent Variable	Whites * (*n* = 153)				Blacks * (*n* = 174)			
	Base Model		Base Model + SES		Base Model		Base Model + SES	
	β (SE)	*p*	β (SE)	*p*	β (SE)	*p*	β (SE)	*p*
BDI **	−0.29 (0.10)	0.003	−0.32 (0.08)	<0.001	−0.05 (0.10)	0.615	0.03 (0.09)	0.723
STAI ***	−0.22 (0.10)	0.037	−0.24 (0.09)	0.010	−0.17 (0.09)	0.069	−0.11 (0.09)	0.205
**PSS**	−0.26 (0.11)	0.017	−0.28 (0.10)	0.005	−0.15 (0.09)	0.090	−0.10 (0.09)	0.279
**CMHS**	−0.12 (0.10)	0.206	−0.14 (0.09)	0.104	−0.27 (0.09)	0.002	−0.22 (0.08)	0.009
Cynicism	−0.20 (0.09)	0.025	−0.22 (0.08)	0.010	−0.26 (0.09)	0.005	−0.22 (0.09)	0.015
Hostile affect	−0.12 (0.10)	0.251	−0.13 (0.10)	0.189	−0.19 (0.09)	0.041	−0.14 (0.09)	0.124
Aggressive responding	−0.00 (0.10)	0.963	−0.02 (0.10)	0.827	−0.21 (0.09)	0.016	−0.18 (0.09)	0.041
Hostile attribution	−0.06 (0.10)	0.551	−0.08 (0.09)	0.364	−0.29 (0.09)	0.001	−0.24 (0.09)	0.006
Social aversion	−0.19 (0.10)	0.070	−0.20 (0.10)	0.045	−0.06 (0.09)	0.485	−0.06 (0.09)	0.503

25(OH)D = 25-hydroxyvitamin D_3_; BDI = Beck Depression Inventory; STAI = State-Trait Anxiety Inventory; PSS = Perceived Stress Scale; CMHS = Cook–Medley Hostility Scale; SES = socioeconomic status. * Serum 25(OH)D was log-transformed and adjusted for age and gender in the base model. BDI was square root-transformed and STAI was log-transformed in the regression. Standardized β coefficients are presented. ** Square root-transformed. *** Log-transformed.
